# Case report of severe myocarditis in an immunocompromised child with Respiratory Syncytial Virus infection

**DOI:** 10.1186/s12887-018-1027-9

**Published:** 2018-02-12

**Authors:** Hiroki Miura, Fumihiko Hattori, Hidetoshi Uchida, Tadayoshi Hata, Kazuko Kudo, Masatoki Sato, Tetsushi Yoshikawa

**Affiliations:** 1 Department of Pediatrics, Fujita Health University School of Medicine, 1-98, Dengakugakubo, Kutsukake-cho, Toyoake, Aichi 470-1192 Japan; 2 Graduate School of Health Sciences, Fujita Health University, Toyoake, Aichi Japan; 3 Department of Pediatrics, School of Medicine, Fukushima Medical University, Fukushima, Japan

**Keywords:** RSV, Myocarditis, Leukemia, Maintenance treatment, Real-time RT-PCR

## Abstract

**Background:**

Respiratory syncytial virus (RSV) infection is common and may be severe among patients with preexisting cardiac anomalies, but direct involvement of myocardial damage is not common in those patients. Additionally, myocardial involvement has been rarely described among immune compromised children.

**Case presentation:**

A 4-year-old girl with acute lymphoblastic leukemia who received maintenance chemotherapy in an outpatient clinic developed systemic inflammatory response syndrome. RSV infection was confirmed by a positive rapid antigen test and serological assay. Subsequently, she was diagnosed with severe myocarditis caused by RSV infection, which was diagnosed by abnormal findings of cardiac echography and ECG and elevated biomarkers for myocardial damage. Then, she was treated in the intensive care unit for 13 days. High amounts of RSV type B RNA was detected in tracheal aspirates and serum sample.

**Conclusion:**

This case report emphasizes that RSV infection may be associated with myocarditis in immunocompromised children receiving maintenance chemotherapy.

## Background

Respiratory syncytial virus (RSV) is one of the most important viral causes of lower respiratory tract infection (LRTI) in infancy and childhood. It is well known that premature infants, infants with cardiac diseases, and immunocompromised patients, including leukemic children, are at high risk for more severe and complicated RSV infection. Although severe LRTI caused by RSV infection is associated with high morbidity in patients with acute lymphoblastic leukemia [1], no RSV myocarditis case has been reported in such patients. This report describes an immunocompromised child with severe myocarditis caused by RSV infection and detection of high amounts of viral genome in not only tracheal tube aspirates but also serum sample based on real-time revers transcription polymerase chain reaction (RT-PCR).

## Case presentation

**Fig. 1:**
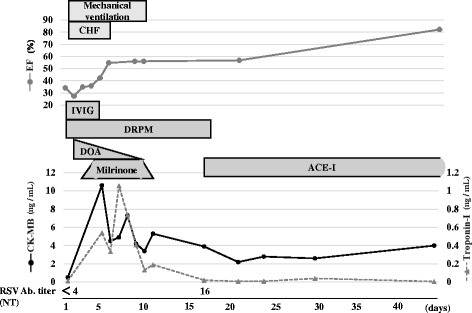
Clinical course of the patient. Kinetics for the ejection fraction and biomarkers of myocardial damage. Myocardial function and myocardial damage were gradually recovered in the acute phase using intensive treatment. CHF: continuous hemofiltration; EF: ejection fraction; CPK: creatine kinase; MB: muscle and brain; ACE: angiotensin converting enzyme inhibitor

A 4-year-old Japanese girl with B-cell acute lymphoblastic leukemia (ALL) presented to the emergency room with febrile convulsion. The age of disease onset was 3 years and the patient received maintenance chemotherapy in our outpatient clinic. Clinical course of patient is shown in Fig. 1. The patient had a high fever (40.1 C°), tachypnea (respiratory rate 38/min), and tachycardia (pulse rate 180/min). She was diagnosed with febrile neutropenia and systemic inflammatory response syndrome (SIRS) based on her neutrophil count, which was 322/μL, as well as remarkably elevated inflammatory responses (CRP: 2.2 mg/dl; procalicitonin: 67.8 ng/mL). Chest X-ray revealed mild perihilar infiltration with normal cardiothoracic ratio (51.6%), and chest computed tomographic scanning showed bilateral consolidation and pleural effusion. Within two hours after admission, the patient’s respiratory condition continued to deteriorate despite oxygen supplementation while lactate levels increased to 62.6 mg/dL. The patient was transferred to the intensive care unit for mechanical ventilation; however, her systolic blood pressure decreased gradually from 116 to 40 mmHg despite hydration and blood transfusion for volume expansion. Therefore, catecholamine administration, intravenous immunoglobulin, and continuous venovenous hemofiltration were initiated. As we suspected myocardial dysfunction, cardiac ultrasound was performed and showed depressed left ventricular function with an ejection fraction of 27%, as well as a left ventricular Tei index of 0.62 indicating global systolic and diastolic ventricular function, mitral regurgitation, and tricuspid regurgitation. An electrocardiogram showed ST-T wave change in V4-6. Furthermore, a marked increase in myocardial markers was noted, including Troponin-I 0.541 ng/mL (normal 0-0.04 ng/mL), myoglobin 91.2 ng/mL (normal 12.8-66.1 ng/mL), and CK-MB 10.6 ng/mL (0.9-5.9 ng/mL).

Two days after admission to the hospital, her systolic blood pressure improved to 120 mmHg and inotropic support was gradually reduced and stopped by day 9. In addition to the administration of milrinone for 11 days, continuous hemofiltration was carried out for 6 days to treat the heart failure. Subsequently, left ventricular function gradually improved, and myocardial markers returned to normal levels 17 days after admission to the hospital. Finally, she was discharged from the intensive care unit on day 13.

Three separate blood cultures were negative. Additionally, the patient tested negative for influenza virus, human-metapneumovirus, adenovirus, and *Group A streptococcus* using rapid antigen tests; however, RSV antigens were detected in a nasal swab at the time of hospital admission. Moreover, seroconversion of RSV antibody titers (< 4 and 16) was confirmed using a neutralizing antibody test (SRL Inc., Tokyo, Japan). In order to elucidate the pathophysiology of RSV infection, serially collected tracheal tube aspirates and serum samples were examined using real-time RT-PCR [2]. Real-time RT-PCR analysis detected high amounts of RSV type B RNA in the tracheal aspirates on day 2 (1.6 × 10^9^ copies/ml) and day 5 (1.4 × 10^8^ copies/ml) after the onset of the disease. Interestingly, viral RNA was also detected in the serum sample obtained on day 1 of the illness (3.2 × 10^4^ copies/ml), but it had decreased to undetectable levels in serum samples collected on days 5 and 8.

In order to examine the pathophysiology of this severe cardiac complication caused by RSV infection, various cytokines and chemokines were measured in serially collected serum samples using the Cytometric Bead Array system (Table 1). Serum samples were serially collected before the onset of the RSV infection and on days 1, 5, 7, and 14 after illness onset. As shown in Table 1, IL-6, IL-10, IL-8, IFN-γ, MCP-1, and IP-10 were markedly elevated at the time of disease onset. The levels of these biomarkers returned to normal by 14 days after illness onset.

**Table 1:** Kinetics of cytokines and chemokines level in serially collected serum samples

Day	Pre	1	5	14	17
IL-2 (pg/mL)	0.0	0.0	0.0	0.0	0.0
IL-4 (pg/mL)	0.0	0.0	0.0	0.0	0.0
IL-6 (pg/mL)	0.0	17,599.1	92.7	0.0	0.0
IL-10 (pg/mL)	0.0	150.6	1.7	0.0	0.0
TNF-α (pg/mL)	0.0	0.0	0.0	0.0	0.0
IFN-γ (pg/mL)	0.0	16.2	64.6	0.0	0.0
IL-8 (pg/mL)	0.0	5376.5	731.4	26.9	20.8
RANTES (pg/mL)	10,878.2	12,011.7	6193.2	17,193.4	20,705.5
MIG (pg/mL)	57.2	141.32	221.7	192.1	114.5
MCP-1 (pg/mL)	238.1	9009.0	667.4	231.0	314.2
IP-10 (pg/mL)	0.0	1104.5	2739.1	404.3	303.0

*IL* interleukin, *TNF* tumor necrosis factor, *IFN* interferon, *RANTES* regulated on activation, normal T cell expressed and secreted, *MIG* monokine induced by interferon g, *MCP* monocyte chemoattractant protein, *IP* interferon g-inducible protein

## Discussion and conclusions

RSV infection in high risk patients, including patients with hematological malignancies, is associated with increased morbidity and mortality. Lymphopenia (lymphocyte counts of < 100/mm^3^) and young age (less than 2 years of age) are predictors of RSV-related LRTI in immunocompromised children [3]. Maintenance treatment is considered to be safer than induction treatment because it is associated with lower levels of immunosuppression. Meanwhile, maintenance treatment carried out in outpatient clinics appears to increase the risk for community acquired RSV infection. Recently, Chu et al. [4] reported that 15/54 (28%) immunocompromised children with RSV infection who were treated in outpatient clinics were later hospitalized due to LRTI. Although RSV-related myocarditis has been demonstrated in healthy children [5–9], to the best of our knowledge, this is the first myocarditis case in association with RSV infection in an immunocompromised patient. Thus, in addition to the LRTI, RSV infection may cause myocarditis in immunocompromised children during maintenance chemotherapy in an outpatient clinic setting.

It is important to note, high amounts of RSV RNA were detected in not only the tracheal aspirates but also the serum sample obtained on day 1 of the illness. A previous study found no association between viral load, including RSV, and disease severity in bronchoalveolar lavage samples collected from hematological stem cell transplant recipients [10]. However, the detection of viral RNA in serum samples was significantly associated with patient mortality [10]. Thus, the detection of RSV RNA in serum samples may serve as a useful prognostic marker in patients with severe RSV infection. Furthermre, in the present case, biopsy of the heart tissue was not performed and thus demonstration of RSV in tissue sample was not possible. This procedure is not routinely performed in children. However, the presence of serum RSV RNA in our patient suggesting systemic viral infection may support the notion of direct invasion of RSV into myocardial tissues as suggested by previous study [6].

It has been suggested that viral invasion of the myocardium or endothelial cells and host immune responses may play important roles in the pathogenesis of viral myocarditis. Several cytokines and chemokines were clearly elevated in our patient during the acute phase of the disease, suggesting that host immune responses may contribute to the pathogenesis of RSV-related myocarditis. It has been suggested that Th1 immune responses initiated by INF-γ [11, 12] and Th1 chemokines (IP-10) [13] may play important roles in the pathogenesis of viral myocarditis. Additionally, in a murine model, MCP-1 was required for the invasion of monocytes/macrophages into the myocardium [14]. Our present findings of elevated cytokines and chemokines are consistent with the previous studies [11–14]. A large number of cases is necessary to elucidate the pathogenesis of myocarditis caused by RSV infection, indeed, this may be difficult to achieve because the incidence of this complication is extremely low. Therefore, in addition to human data, an animal model should improve our understanding of the precise pathological mechanisms of RSV-related myocarditis.

In conclusion, herein, we reported on a 4-year-old Japanese girl with ALL who suffered from severe myocarditis associated with RSV infection during maintenance treatment. RSV type B RNA was detected in both tracheal aspirates and serum samples during the acute phase of the disease. This case report emphasizes that in addition to lower respiratory tract infections, RSV infection may cause myocarditis in immunocompromised children receiving maintenance chemotherapy.

## Abbreviations

ALL: Acute lymphoblastic leukemia
CK: Creatine kinase
CRP: C-Reactive Protein
ECG: Electrocardiogram
IFN: Interleukin
IL: Interleukin
IP: Interferon g-inducible protein
LRTI: Lower respiratory tract infection
MB: Muscle and brain
MCP: Monocyte chemoattractant protein
RNA: Ribonucleic acid
RSV: Respiratory syncytial virus
RT-PCR: Real-time revers transcription polymerase chain reaction
